# Accelerometers Identify New Behaviors and Show Little Difference in the Activity Budgets of Lactating Northern Fur Seals (*Callorhinus ursinus*) between Breeding Islands and Foraging Habitats in the Eastern Bering Sea

**DOI:** 10.1371/journal.pone.0118761

**Published:** 2015-03-25

**Authors:** Brian C. Battaile, Kentaro Q. Sakamoto, Chad A. Nordstrom, David A. S. Rosen, Andrew W. Trites

**Affiliations:** 1 Marine Mammal Research Unit, Fisheries Centre, University of British Columbia, Aquatic Ecosystems Research Laboratory, Vancouver, British Columbia, Canada; 2 Graduate School of Veterinary Medicine, Hokkaido University, Sapporo, Hokkaido, 060–0818, Japan; Institut Pluridisciplinaire Hubert Curien, FRANCE

## Abstract

We tagged 82 lactating northern fur seals (*Callorhinus ursinus*) with tri-axial accelerometers and magnetometers on two eastern Bering Sea islands (Bogoslof and St. Paul) with contrasting population trajectories. Using depth data, accelerometer data and spectral analysis we classified time spent diving (30%), resting (~7%), shaking and grooming their pelage (9%), swimming in the prone position (~10%) and two types of previously undocumented rolling behavior (29%), with the remaining time (~15%) unspecified. The reason for the extensive rolling behavior is not known. We ground-truthed the accelerometry signals for shaking and grooming and rolling behaviors—and identified the acceleration signal for porpoising—by filming tagged northern fur seals in captivity. Speeds from GPS interpolated data indicated that animals traveled fastest while in the prone position, suggesting that this behavior is indicative of destination-based swimming. Very little difference was found in the percentages of time spent in the categorical behaviors with respect to breeding islands (Bogoslof or St. Paul Island), forager type (cathemeral or nocturnal), and the region where the animals foraged (primarily on-shelf <200m, or off-shelf > 200m). The lack of significant differences between islands, regions and forager type may indicate that behaviors summarized over a trip are somewhat hardwired even though foraging trip length and when and where animals dive are known to vary with island, forager type and region.

## Introduction

Quantifying the time an animal spends in various activities such as locomotion, resting, and foraging can be used to estimate energy expenditure and understand life history strategies. In the case of northern fur seals (*Callorhinus ursinus*), determining activity budgets could help to explain why the population trajectories of two eastern Bering Sea populations differ. The population of fur seals on St. Paul Island (central Bering Sea) has declined since 1998 at an annual rate of 5.5%, while the population on Bogoslof Island (southern Bering Sea) has increased at an annual rate of 11.7% since 1997 [1–3]. Unfortunately, at-sea observations of fur seal behavior are difficult to obtain.

Telemetry devices carried by northern fur seals since the 1990’s have provided at-sea locations of individuals and recordings of swimming depths by time of day [4,5]. TDR (Time-Depth-Recording) data have also been augmented with acoustic data to differentiate behaviors (locomotion, diving, resting and surface activity) and flipper stroke rates associated with different types of dives [6]. Such telemetry has revealed some differences in foraging habitat and foraging behavior between lactating fur seals breeding at different sites [4,5,7–9]. Most notably, they have shown that fur seals from Bogoslof Island forage almost exclusively in deep basin waters with very little diving occurring during the day, while individuals from St. Paul Island forage over both the shelf and in deep basin waters [9,10]. In addition, they have revealed that some St. Paul animals make substantial numbers of dives during the day—a behavior that is highly associated with animals that forage over the continental shelf [10,11].

The current breakdown of at-sea behaviors of northern fur seals is relatively coarse, and fails to differentiate surface activities. One means to document more specific behaviors is by categorizing movements recorded by tri-axial accelerometers [12–19]. Identifying episodes of feeding, locomotion and other behaviors can be useful in basic physiological studies, bioenergetic modeling, as well as ecological studies identifying foraging hotspots, informing predator prey dynamics, and marine resource management [20–24].

Our study sought to expand the number of previously differentiable behaviors of northern fur seals by analyzing acceleration data recorded during foraging trips. Data were collected from lactating females from St. Paul and Bogoslof Islands using a biologging tag that included tri-axial accelerometers (Daily Diary tag, Wildlife Computers, Redmond, WA). Our goals were to 1) differentiate between common fur seal behaviors, 2) create an at-sea activity budget, and 3) make ecologically relevant comparisons both within and between the two island populations of northern fur seals. We also validated our interpretation of some of the accelerometry data from fur seals in the field by filming instrumented female fur seals in captivity.

## Methods

### Ethics Statement

All procedures were conducted under the National Oceanic and Atmospheric Administration permit no. 14329 and the protocol was approved by the Committee on Animal Care at the University of British Columbia (permit no. A09–0345).

### Tagging

**Fig 1:**
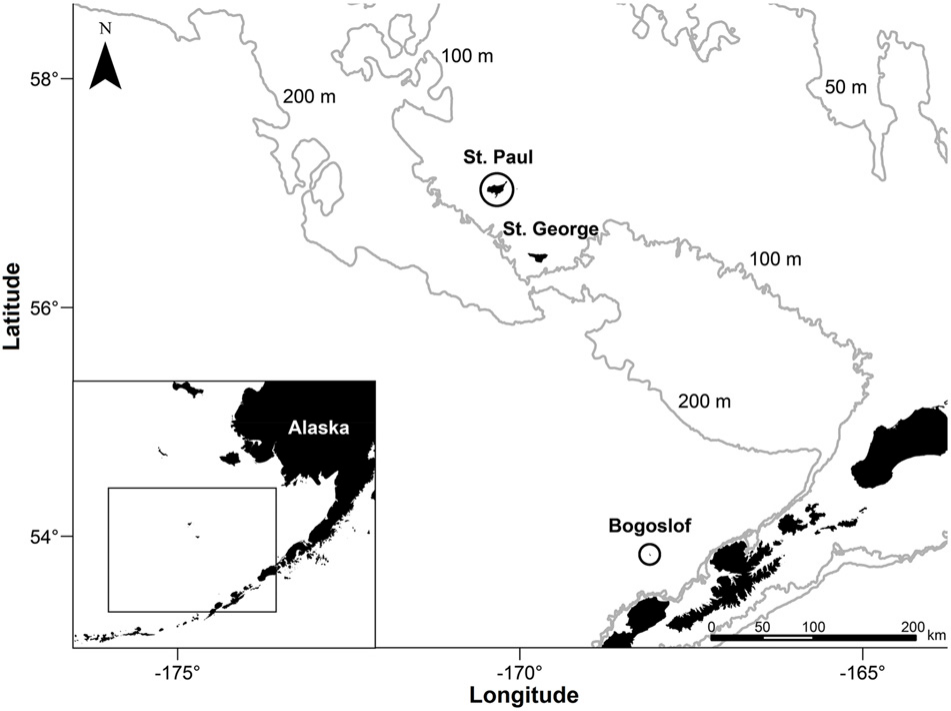
Study sites in the eastern Bering Sea. Northern fur seals were tagged for this study on St. Paul Island and Bogoslof Island. Also shown are the 50, 100 and 200m depth contours.

From July 10^th^ to September 19^th^ 2009, we tagged lactating northern fur seals on St. Paul Island (in the Pribilof archipelago) and on Bogoslof Island (a volcanic pinnacle south of the Pribilof Islands and just north of the Aleutian Islands chain) (Fig. 1). Fur seals were tagged with a combination of Wildlife Computers Mk10-F (with Fastloc GPS), first-generation Daily Diary tags, and VHF transmitters. Both Wildlife Computers tags are about the size of a deck of cards and were mounted dorsally along the centerline of the animal between the shoulder blades. Seals were recaptured between 3 and 28 days after deployment to recover the tags. Further details of the capture, recapture and tagging methodology can be found in Nordstrom et al. [10].

### Behavior categorization

Our behavior data were collected using the Daily Diary tag. This tag had tri-axial accelerometers and magnetometers that recorded acceleration and the earth’s magnetic field in the 3 spatial dimensions (from the animal’s frame of reference, anterior-posterior (surge or x-axis), dorsal-ventral (heave or z-axis) and lateral (sway or y-axis)). It also recorded swimming depth, internal temperature of the tag, and conductivity to indicate wet/dry. The acceleration and magnetic field sensors were set to collect data at 16Hz, while the remaining sensors recorded every second (1Hz).

**Fig 2:**
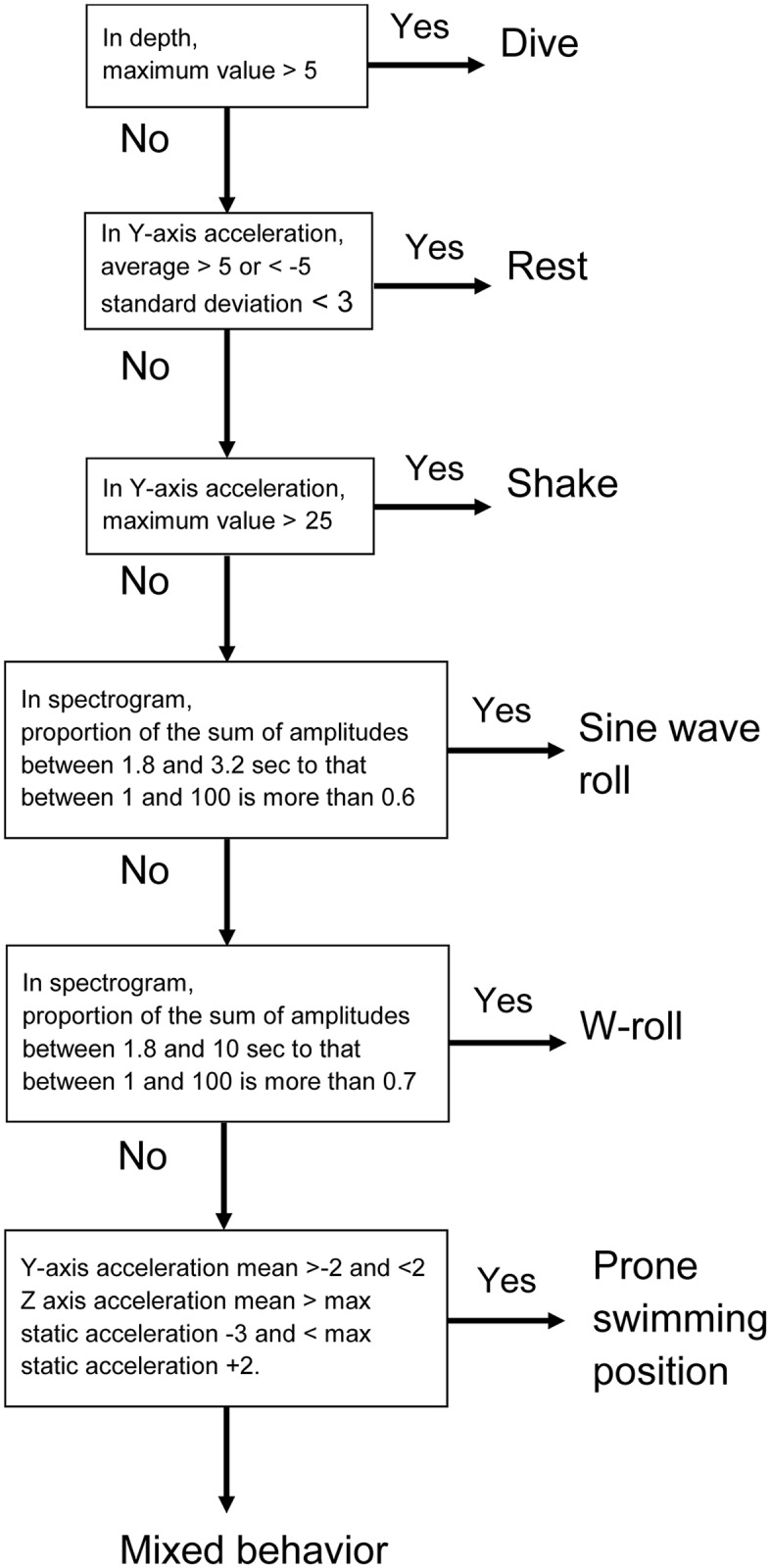
Behavior algorithm flow chart. Flow chart for the algorithm used to define the 6 northern fur seal behaviors with the Igor Pro Ethographer software and R software.

Behavioral analysis was conducted using the depth channel, the lateral accelerometer, and the dorso-ventral accelerometer. Behavioral analysis occurred in two stages. The first stage involved importing the data into Igor Pro wave analysis software (WaveMetrics). The Ethographer [14] software add-in was initially used to identify diving behavior based on dive depths. It was then used to identify commonly occurring, repeated signals in the lateral acceleration data using spectral analysis. These repeated signals (behaviors) were differentiated into 4 distinct movement patterns—subsequently identified as resting, shaking (also identified with pelage grooming) and two types of surface rolling (“sine wave” and “W”) (Fig. 2). Remaining time was placed into an undifferentiated “mixed” category. Analysis occurred over 1 minute bins, and the dominant behavior was assigned to each period. The second stage of the behavioral analysis involved importing the data into R [25] and visually examining the accelerometry data to identify a 6^th^ behavioral category from the “mixed” category, which we termed “prone position” behavior.

“Diving”, the first behavior we differentiated, was identified using the depth channel and defined as excursions below a depth of 5m during the day and below a depth of 0 meters during the night. This definition was primarily designed to distinguish between diving that was most likely foraging, and diving that was most likely other sub surface behaviors (which vary between day and night). We defined night as beginning and ending at nautical twilight, which is a solar elevation angle of 6 degrees below the horizon. Given that night dives tend to be shallow and short, we assigned diving behavior to a given minute if the animal was below the surface for more than 25% of the time in that minute. This definition differs from our “dominant behavior” designation for all other behaviors, and underestimates if diving occurred during a minute but overestimates the overall time an animal was actually diving.

Myctophids are a deep-water species and prey item for northern fur seals from Bogoslof and St. Paul Island [26,27]. These species migrates vertically to the near-surface waters during the night, where it is consumed by fur seals and sea birds. Parades et al. [28] found myctophids to be present in ~90% of sampled Black-legged kittiwakes (*Rissa tridactyla*), which are plunge divers that are unable to dive below the top meter of water. It is thus possible for fur seals to feed on myctophids while making very short shallow dives—hence our need to establish criteria to capture this relatively brief but important foraging behavior.

We differentiated the remaining time into exclusive behavioral categories using the lateral and dorsal-ventral axes of accelerometer data. Raw data from the accelerometer channel was in mV, with a reading of ~10 mV roughly coinciding with the force of gravity at 9.8 m s^-2^. We defined resting behavior using a two-step criteria: 1) average lateral acceleration > 5 m s^-2^ or < -5 m s^-2^ with 2) a standard deviation < 3 m s^-2^. These conditions indicated that the fur seals were laying on either lateral side at the water surface and moving very little. Shaking behavior was defined when bursts of strong lateral acceleration (> ±25 m s^-2^) were recorded.

We identified and defined other behaviors in the accelerometer data based on commonly occurring waves with consistent amplitude and frequency. Frequency analysis can be used to identify cyclical oscillation waves. We thus extracted rolling behaviors from the acceleration data by employing a frequency analysis method based on continuous wavelet transformation with a Morlet mother wavelet as described by Sakamoto et al. [14]. This method enabled us to extract the times of cyclic oscillation that had specific periodicities. The spectrogram examined the periodicity in 8 steps corresponding to 1.0, 1.8, 3.2, 5.6, 10, 18, 32, and 56 seconds, and showed a strong signal between 2–3 seconds corresponding to the periodicity of the rolling behaviors. We then calculated the proportion of the sum of the signal intensity at the 1.8 and 3.2 second cycles to the sum of all cycles where a high proportion indicated that the shape of the acceleration signal was similar to a sine wave. We defined the basic sine wave rolling behavior when this proportion was more than 60%. We also calculated the sum of the signal intensity at the 1.8, 3.2, 5.6 and 10 second cycles and we defined the behavior as a W-roll when the sum of these proportions was greater than 70%. *K*-means clustering (an unsupervised classification algorithm) was then used to aggregate the behavior spectra over each second by minimizing the within-cluster sum of squared Euclidean distances from the cluster (behavior) centroids, thereby determining the dominant behavior.

While we were unable to quantify swimming effort using flipper strokes, visual inspection of the accelerometer channels indicated that the animals did spend considerable time at the surface in a prone position. This appeared to occur most often near the end of a trip and was hypothesized to indicate swimming. Seals lying on their stomachs in the prone position have a static acceleration of ~0 on the sway axis and ~10 on the heave-axis accelerometer, with some minor variation between tags due to sensor variability. We thus defined an animal to be in the prone position when the mean of the sway-axis acceleration was 0 (± 2) and the mean of the heave-axis acceleration was 10 (+2,-3). This slight negative skewing of the heave-axis acceleration reflected our observation that some individuals tended to roll slightly when in the prone swimming position.

### Filmed captive behavior

We filmed a group of captive fur seals (4.5 yr old females at the Vancouver Aquarium, Canada) for approximately 18.5 hours over 7 days between May and December 2012 to validate the at-sea behavior our algorithm identified from the accelerometry data of the wild fur seals. One of the females in the group (NF08ME) was fitted with a specially-designed harness with an attached accelerometer. The instrumented fur seal swam freely with conspecifics in a 15 × 15 × 3.5 m deep research pool for several hours at a time, while we filmed her behavior with a high definition camera mounted above the pool. The anterior-posterior axis of acceleration was used to identify the porpoising behavior.

### Speed analysis

Speeds were calculated between GPS fixes (obtained from the Mk-10F tag) via simple point interpolation and dividing distance traveled by time. GPS points associated with unrealistic travel speeds of greater than 3 m s^-1^ were removed. We then assigned speed to all behaviors exhibited between GPS fixes and average speeds per behavior were calculated on a per trip basis. We then used mixed effects models (with individual fur seal as a random effect) to determine if specific behaviors tended to have faster traveling speeds associated with them. We investigated two models (stratified by island as fur seals from St. Paul tended to have more GPS fixes per time than those from Bogoslof) where the basic model took the form of *Speed*_*i*_ = *β*_*j*_ *Behavior*_*j*_ *+ seal*_*k*_ *b*_*k*_ + *e*_*i*_, where *b*_*k*_ and *e*_*i*_ are both normally distributed random variables.

### Behavior analyses

We sought to determine if there was a difference in the proportion of behaviors exhibited by different subsets of fur seals, and whether behaviors changed based on the length of the foraging trip. We were also interested whether differences existed between the two islands, between animals on St. Paul that used the on-shelf or off-shelf habitats, and between nocturnal or cathemeral foragers (“cathemeral “defined by Nordstrom et al. [10] for animals with > 10% of their dives during the day).

The proportions of each behavior exhibited by fur seals during a foraging trip always summed to one. Such data have some unusual statistical properties in that the proportions are highly correlated (e.g., if diving takes up 50% of the time, there is only 50% of the time that can be allocated to the other 6 behaviors) and the proportions are bounded by 0 and 1. This is a classic “compositions” data set, yet popular methods to analyze these data are not necessarily intuitive and involve taking log ratios of proportions before statistically comparing the ratios. There are at least three 3 R packages available for this type of analysis in addition to numerous textbooks and papers that vastly range in their accessibility for biologists. We relied heavily on van den Boogaart and Tolosana-Delgado [29], while the more mathematically inclined may prefer Aitchison [30]. We primarily used the package Compositions for data exploration and visualization. For the statistical analyses, we used fractional multinomial logistic regression using the fmlogit package in STATA, which uses the untransformed proportions themselves and accounts for multiple observations per animal (as many of our animals took more than one foraging trip). The full model for the fractional multinomial logistic regression included trip date, trip length, island (Bogoslof or St. Paul), forager type (nocturnal or cathemeral) and foraging region (on-shelf or off-shelf). Based on the results of this model, we used a simple linear regression to characterize the relationship between total diving time over the course of a day and total nighttime available.

## Results

### Tagging

Tags from seals on Bogoslof Island (n = 41 individuals) logged 111 useable foraging trips, while those from seals on St. Paul Island (n = 41) recorded 51 foraging trips. Trip duration for the Bogoslof seals averaged 3.3 days ranging from 3.75 hours to just over 13 days, while trip duration for St. Paul Island seals averaged 7.7 days ranging from just over 4 days to nearly 17 days. Trip distance as calculated by interpolation between GPS locations averaged 189 km and ranged from 0.5 to 525 km, while St. Paul Island trips averaged 582 km and ranged from 243 to 1265 km.

### Behavior categorization

**Fig 3:**
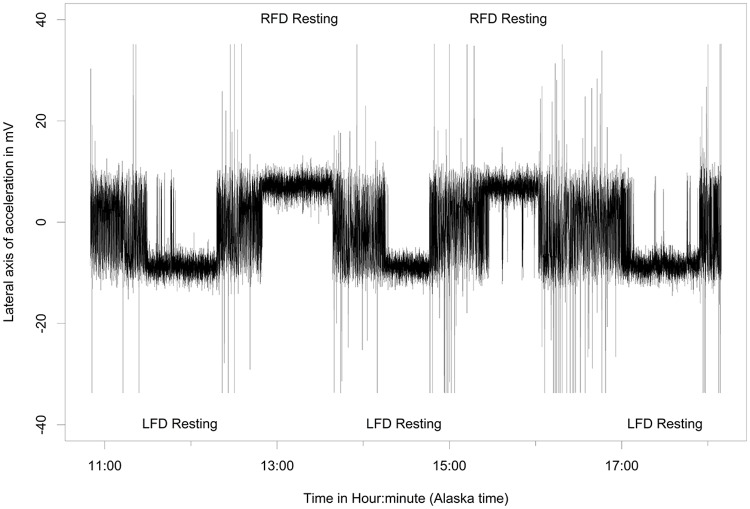
Resting accelerometer signal. Five periods of resting behavior punctuated with active periods recorded at-sea during ~7 hours from a lactating northern fur seal. Resting behavior is easily recognizable as periods with low variability in the lateral axis accelerometer signal with magnitudes near that of gravity. Note that the data show the animal resting for three periods on one side of her body, followed by two periods turned over on the other side.

**Fig 4:**
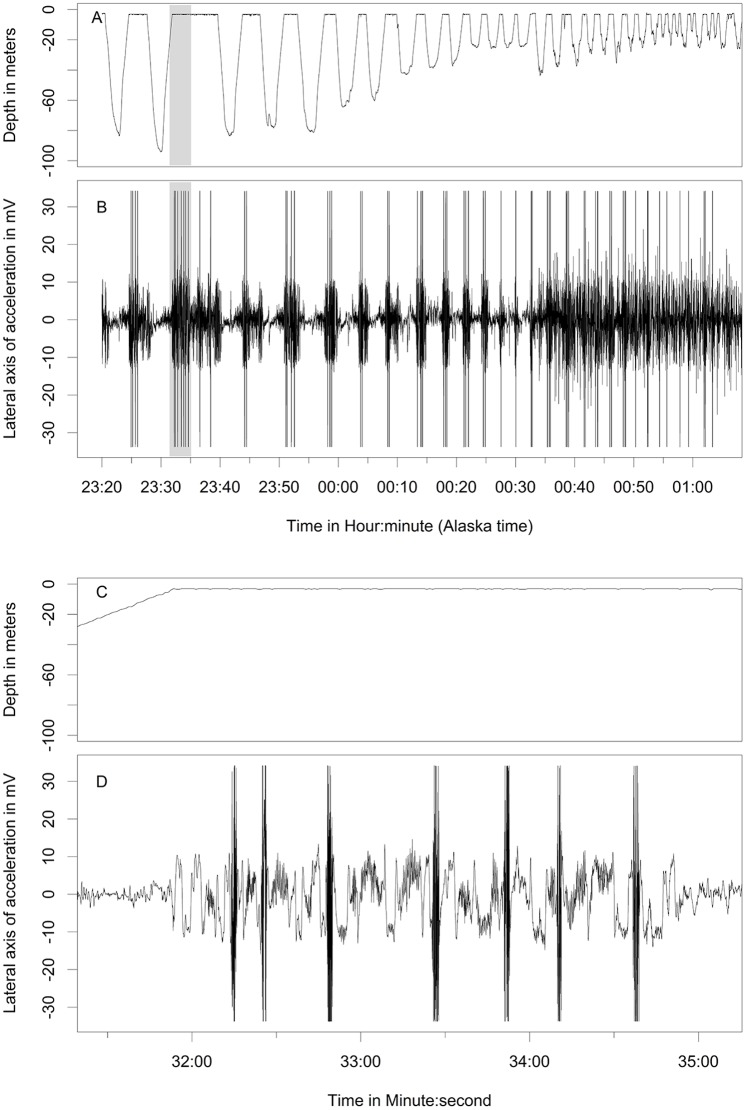
Shaking and grooming accelerometer signal. Shaking behavior of a lactating northern fur seal indicated by the brief signal in the accelerometer values that reach beyond-30 and 30 indicating that the forces were greater than the maximum and minimum recordable by the instruments. The top two panels (A and B) show a common time that shaking occurred between foraging dives, and the bottom two panels (C and D, an expansion of the shaded area in panels A and B) show a detail of this pattern of shaking that was often associated with grooming where the animal spends a few seconds on one side, rolling over on the other for a few seconds, and periodically shaking.

**Fig 5:**
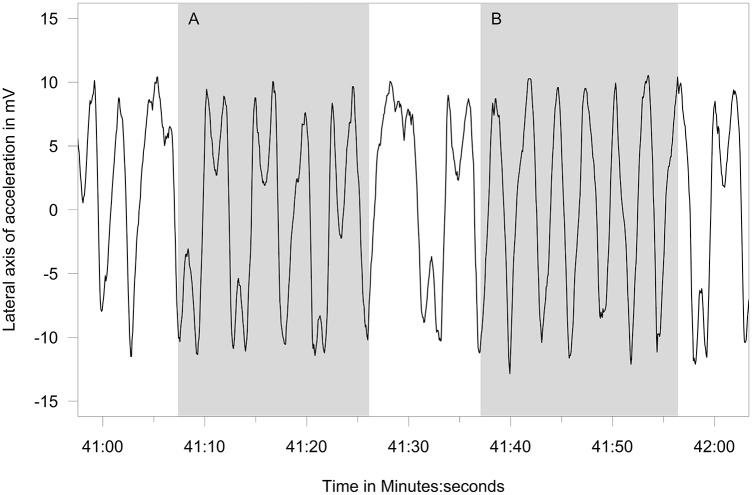
Surface rolling accelerometer signal. Lateral axis accelerometer showing the two types of surface rolling behavior of a lactating northern fur seal—the W-roll (A) and the full 360° roll we have termed the Sine-roll (B).

**Fig 6:**
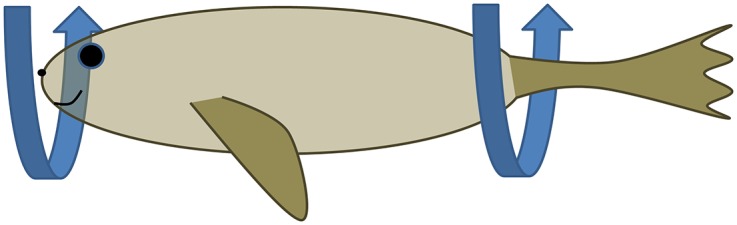
Schematic of surface rolling behavior. The W-roll consists of the fur seal turning ~300° in one direction, then turning back ~300° in the other direction while the sine-roll consists of a constant turning on one direction.

**Fig 7:**
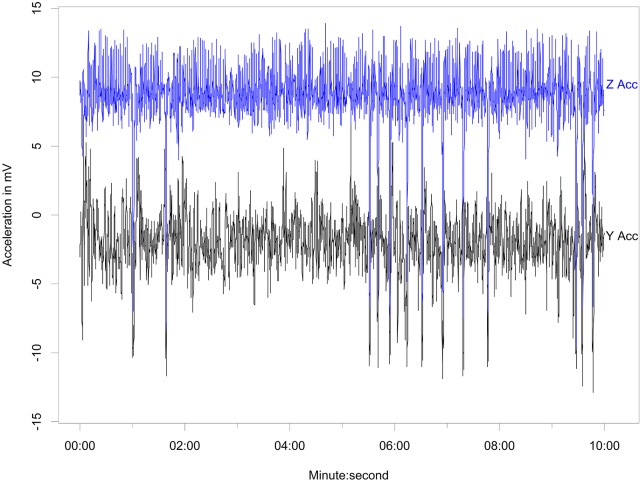
Prone position accelerometer signal. Traces of the Y (lateral) and Z (dorso-ventral) axes of acceleration indicating an animal in the prone position with ~13 instances of rolling over a 10 minute period.

**Fig 8:**
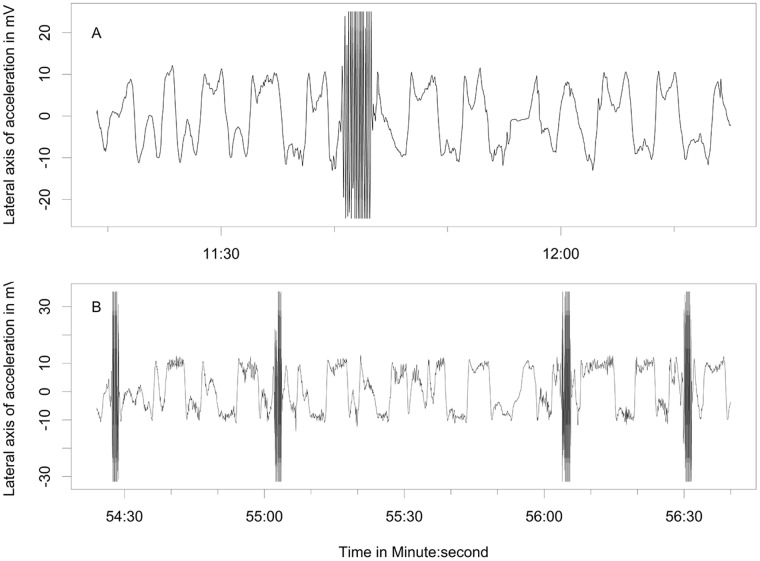
Captive behavior accelerometer signal. Lateral axis accelerometer showing W-roll behavior (with a single shaking event) (A) and the shaking and grooming of the pelage behavior (B) in a captive northern fur seal.

**Fig 9:**
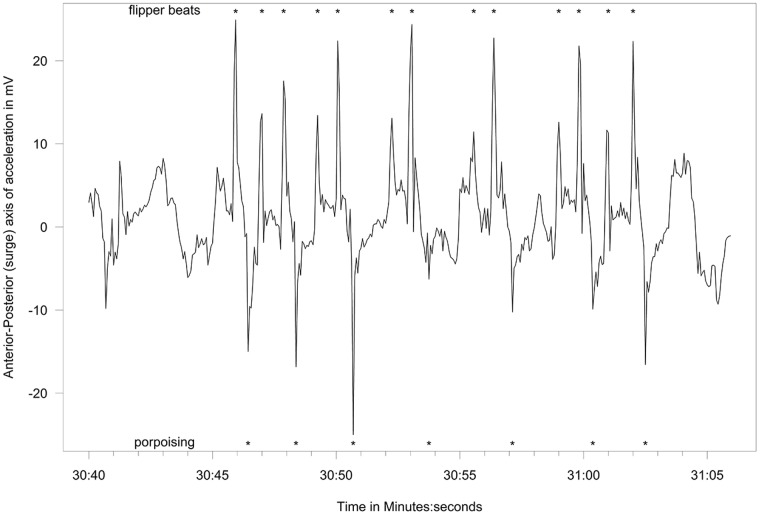
Captive porpoising accelerometer signal. Trace of the Anterior-Posterior axis accelerometer of a female fur seal in captivity. Positive spikes marked with * indicate peak forward acceleration of a flipper beat while negative spikes indicate peak deceleration from porpoising. Peaks above |20| indicate acceleration forces more than double the force of gravity.

Behaviors differentiated from the free-ranging fur seals using acceleration data included resting (Fig. 3), shaking/pelage grooming (Fig. 4), the two types of rolling behavior, the W-roll (based on the wave form) and a sine wave-roll (Figs. 5, 6), and the prone position (Fig. 7). The data indicated the animals spent an unexpectedly large amount of time engaged in the two rolling behaviors (Table 1). Observations of the captive animals confirmed that we had correctly identified the rolling behavior (Fig. 8A) and shaking/grooming (Fig. 8B), as well as successfully identifying and characterizing porpoising behavior (Fig. 9). Thus, we are confident in our ability to interpret these specific behavioral categories represented by changes in acceleration.

**Table 1:** Mean proportions of time engaged in specific behaviors at-sea by lactating northern fur seals from Bogoslof and St. Paul islands as determined from acceleration signals and depth sensors.

Variable	Behavior															
	Dive		Rest		Shaking/Grooming		Rolling						Mixed		Prone	
							Sine		W		Total					
Bogoslof	0.292	(0.10)	0.072	(0.06)	0.096	(0.06)	0.047	(0.05)	0.227	(0.11)	0.274	(0.13)	0.155	(0.09)	0.111	(0.12)
St. Paul	0.284	(0.07)	0.081	(0.04)	0.073	(0.02)	0.086	(0.07)	0.245	(0.10)	0.331	(0.11)	0.143	(0.07)	0.087	(0.09)
*Both Islands*																
Cathemeral	0.295	(0.07)	0.087	(0.04)	0.084	(0.03)	0.060	(0.03)	0.261	(0.09)	0.321	(0.09)	0.146	(0.06)	0.067	(0.06)
Nocturnal	0.288	(0.10)	0.073	(0.06)	0.089	(0.05)	0.060	(0.06)	0.228	(0.11)	0.287	(0.13)	0.152	(0.08)	0.110	(0.11)
Off-shelf	0.291	(0.10)	0.074	(0.06)	0.090	(0.05)	0.057	(0.06)	0.227	(0.11)	0.283	(0.13)	0.153	(0.09)	0.109	(0.11)
On-shelf	0.284	(0.06)	0.080	(0.04)	0.082	(0.02)	0.074	(0.04)	0.263	(0.10)	0.336	(0.10)	0.142	(0.06)	0.076	(0.08)
*St*. *Paul only*																
Cathemeral	0.295	(0.07)	0.087	(0.04)	0.084	(0.03)	0.060	(0.03)	0.261	(0.09)	0.321	(0.09)	0.146	(0.06)	0.067	(0.06)
Nocturnal	0.274	(0.07)	0.077	(0.04)	0.065	(0.02)	0.108	(0.08)	0.232	(0.11)	0.340	(0.12)	0.141	(0.08)	0.104	(0.11)
Off-shelf	0.284	(0.08)	0.083	(0.05)	0.064	(0.03)	0.101	(0.08)	0.225	(0.10)	0.326	(0.11)	0.145	(0.08)	0.099	(0.10)
On-shelf	0.284	(0.06)	0.080	(0.04)	0.082	(0.02)	0.074	(0.04)	0.263	(0.10)	0.336	(0.10)	0.142	(0.06)	0.076	(0.08)

Proportions are averaged over foraging trips and stratified by island, forager type (cathemeral or nocturnal), and region (on or off-shelf). Standard deviations are in parenthesis.

Diving was the most common behavior across any of the stratifications at just under 30% (Table 1), followed closely by the W-roll, resting, shaking/grooming and the prone position, which each constituted between 5 and 10% of the daily behavior. The sine-roll was the least exhibited behavior, though if combined with the W-roll (29%) was greater than the proportion of time spent diving (30%). Finally, the mixed, or unknown, category constituted about 15% of the fur seal activity budget.

### Behavior analyses

**Fig 10:**
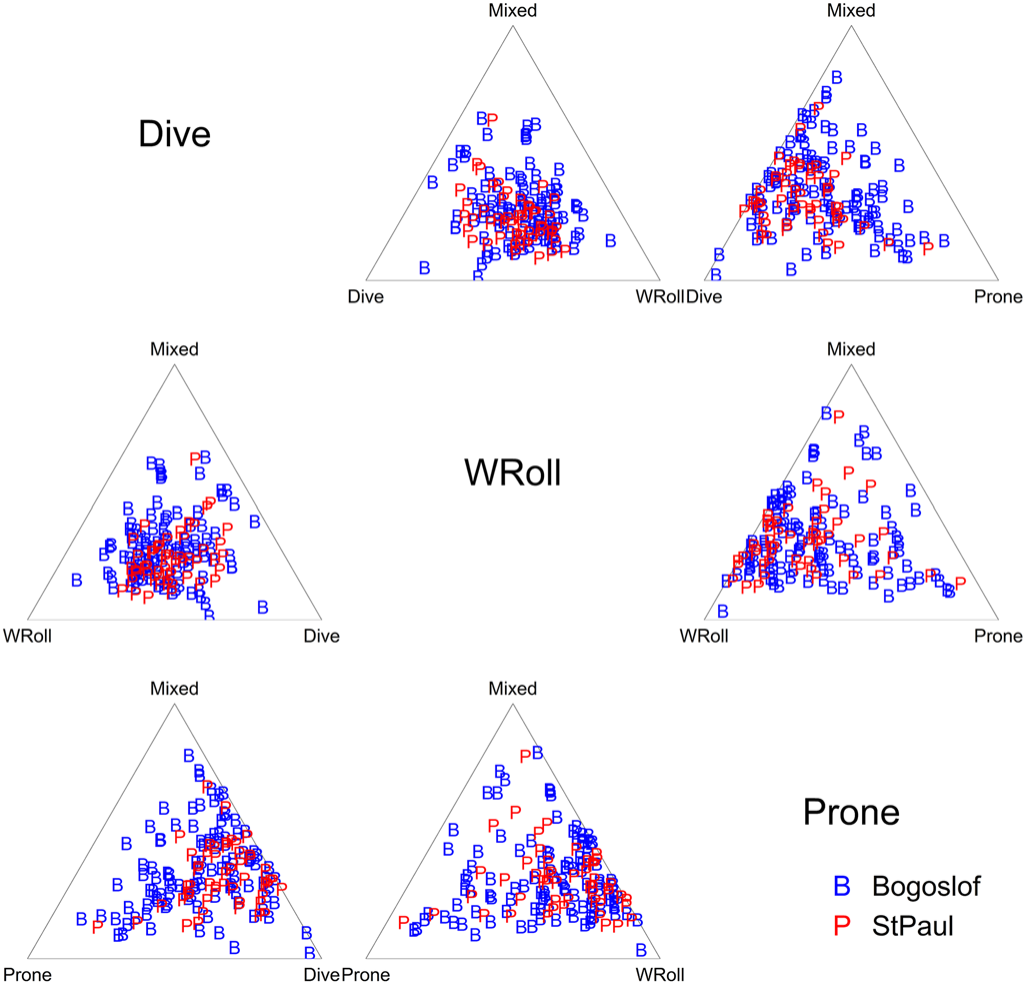
Behavior ternary matrix for different islands. Ternary matrix for 4 behaviors with the “mixed” behavior present in each ternary diagram. Data in each diagram is plotted with the three categories summing to 100% of the proportion, where plots close to the corners indicate high percentages of time allocated to the behavior labeled at that corner, while data near the side directly opposite that corner indicates low percentages of time allocated to that behavior. Proportions of each behavior are color and symbol coded by Island. Note that plots exhibiting different groupings by explanatory variable would indicate potential significant differences, but that there is little differentiation in clumping of proportions by island. (See also S1 Fig. that includes all 7 behaviors and S2–S3 Figs. that examine patterns by foraging region and cathemeral/nocturnal explanatory variables).

**Fig 11:**
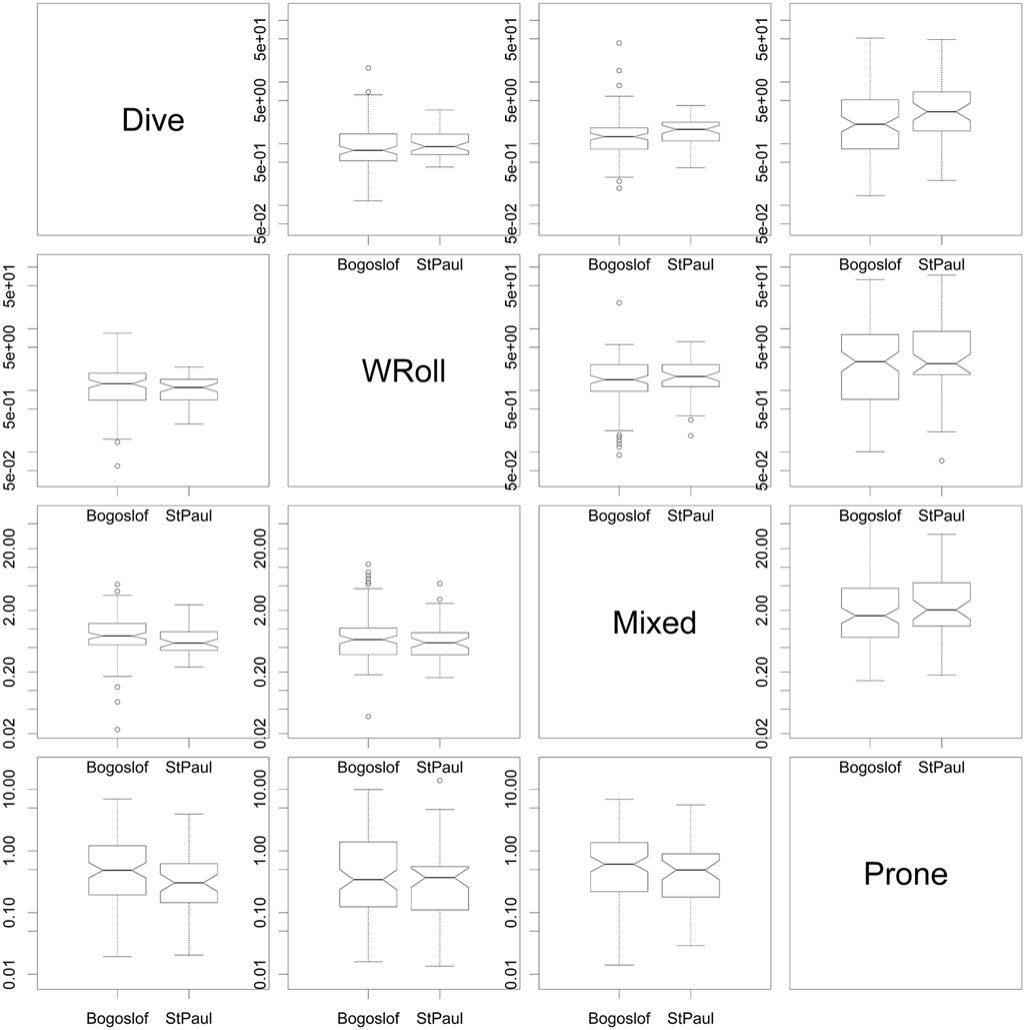
Behavior boxplot matrix for different islands. Boxplot matrix of behavior log ratios for the 4 behaviors plotted in Fig. 10. Note that non-overlapping notches in the two box plots would indicate potential for significant behavioral differences, but that most of the notches overlap. (See also S4 Fig. that includes all 7 behaviors and S5–S6 Figs. that examine behaviors by foraging region and cathemeral/nocturnal explanatory variables).

**Fig 12:**
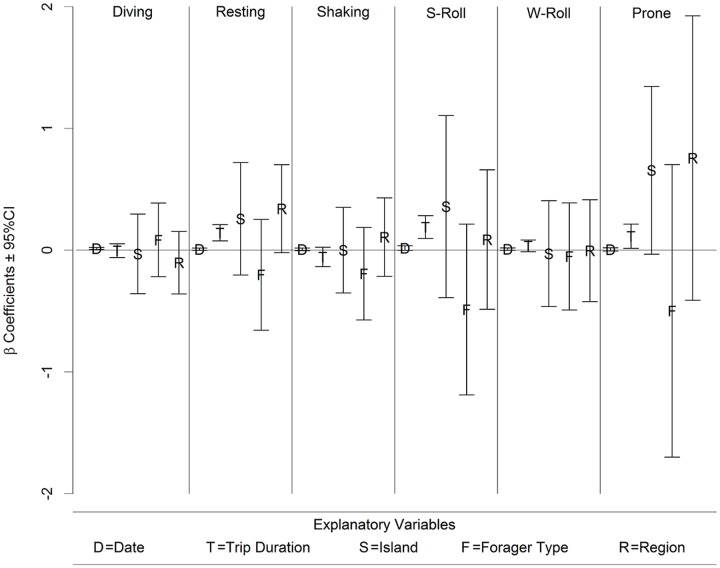
Fractional multinomial logistic model parameters. Parameter estimates for the most complicated model including island, region, forager type and trip duration. Note that only four of the coefficient’s confidence intervals do not include 0.

**Fig 13:**
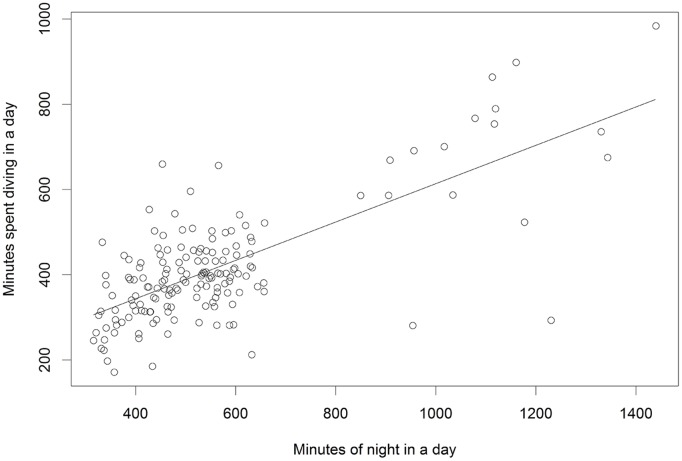
Relationship between total diving time and night time. Linear regression indicating that the number of minutes of darkness in a day is positively related to the number of total minutes of diving during a day.

On the whole, there does not seem to be any indication that the fur seal behaviors differ between any of our explanatory variables. The ternary diagrams do not show separation of proportions by explanatory variables (Fig. 10, see S1–S3 Figs. for the full suite of behaviors), and the vast majority of box plots show overlap on the notches (Fig. 11 and S4–S6 Figs.). Similarly, the lowest AIC_c_ indicates the best model is simply the constant with only four coefficients of 30 being significantly different from 0 in the most complicated model (Fig. 12, Table 2), one of which is the effect of date on diving. Further examination indicated that total diving time in a day increased with total night time available (as the summer progressed) (n = 162, slope = 0.45, t = 13.1, p<0.001) (Fig. 13).

**Table 2:** Fractional multinomial logistic regression models investigated.

Model Parameters	K	Log pseudolikelihood	AIC_c_	Δ AIC_c_
*Constant*	6	-283.7	579.9	0.0
*Trip Duration + Constant*	12	-282.0	590.1	10.2
*Island + Constant*	12	-283.0	592.1	12.2
*Date + Constant*	12	-283.1	592.3	12.4
*Forager Type + Constant*	12	-283.4	592.9	13.0
*Region + Constant*	12	-283.4	593.0	13.1
*Lunar Phase + Constant*	12	-283.4	593.0	13.1
*Trip Duration + Island + Constant*	18	-281.4	603.7	23.8
*Trip Duration + Forager Type + Constant*	18	-281.4	603.8	23.9
*Trip Duration + Region + Constant*	18	-281.6	604.2	24.3
*Island + Forager Type + Constant*	18	-282.6	606.2	26.3
*Island + Region + Constant*	18	-282.8	606.6	26.7
*Forager Type + Region + Constant*	18	-283.2	607.4	27.5
*Trip Duration + Island + Forager Type + Constant*	24	-281.1	619.2	39.3
*Trip Duration + Island + Region + Constant*	24	-281.3	619.6	39.7
*Trip Duration + Forager Type + Region + Constant*	24	-281.3	619.7	39.8
*Island + Forager Type + Region + Constant*	24	-282.6	622.2	42.3
*Trip Duration + Island + Forager Type + Region + Constant*	30	-281.0	636.7	56.8

Number of model parameters (K), Akaike Information Criterion corrected for small sample size (AIC_c_) and the difference in AIC_c_ between the best model (ΔAIC_c_).

### Speed analysis

**Fig 14:**
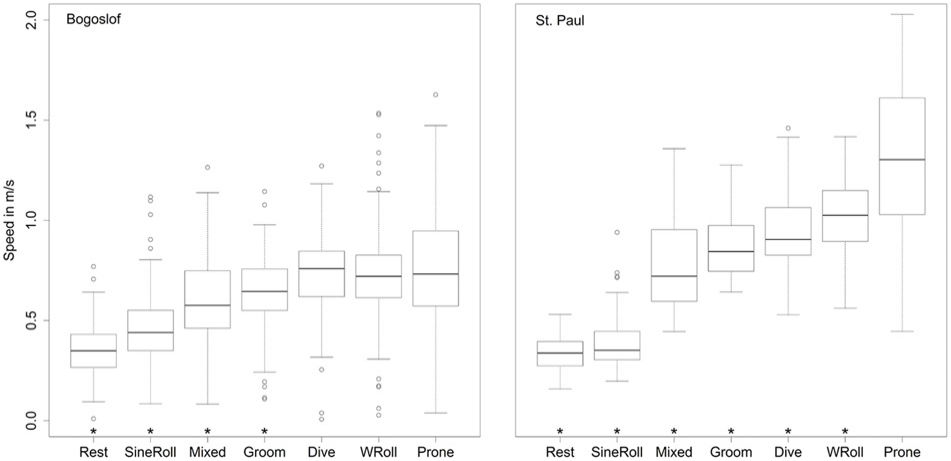
Swimming speeds associated with behaviors. Box plots of the speeds calculated from GPS locations associated with the seven behavioral states. * indicate the behavioral speeds were significantly different from the Prone position speed as determined from mixed effects models where animal was a random effect (n = 117 for Bogoslof and n = 51 for St. Paul).

The comparison of GPS calculated speeds for each of the behaviors (Table 3) indicated that the prone position was significantly faster than all other behaviors from St. Paul Island and most of the other behaviors from Bogoslof Island. However, it was not significantly different from the diving and W-roll behavior for animals from Bogoslof (Fig. 14). Coefficient values (Table 3) are changes in speed relative to the prone position; hence the prone position coefficient is the mean speed for the prone position behavior.

**Table 3:** Parameter estimates for the effect of behavior on travel speeds.

Bogoslof	β-value	t-value	p-value
*Prone*	0.738	29.27	<0.001
*Dive*	-0.022	-0.75	0.451
*Resting*	-0.387	-12.59	<0.001
*Shaking/grooming*	-0.107	-3.65	<0.001
*Sine-roll*	-0.257	-8.19	<0.001
*W-roll*	-0.017	-0.58	0.560
*Mixed*	-0.141	-4.76	<0.001
Pribilofs			
*Prone*	1.303	38.31	<0.001
*Dive*	-0.363	-8.73	<0.001
*Resting*	-0.953	-22.90	<0.001
*Shaking/grooming*	-0.414	-9.95	<0.001
*Sine-roll*	-0.897	-21.56	<0.001
*W-roll*	-0.280	-6.72	<0.001
*Mixed*	-0.504	-12.13	<0.001

The Prone behavior was used as the relative parameter so that the β’s for the other behaviors are adjustments to the Prone β and significance values indicate that the β’s are significantly different from 0 but can be interpreted as significantly different speeds from the Prone behavior speed.

## Discussion

Using spectral analysis on accelerometer data to categorize behavior is a relatively easy way to discover, characterize, and distinguish behavioral types relative to other methods currently available. Not only are there relatively inexpensive software programs readily available for implementation, but the method does not require sophisticated statistical analyses common to other methods [31–33], thereby making spectral analysis readily accessible to most biologists. In our case, it allowed us to construct an activity budget for lactating northern fur seals. We were also able to validate our behavioral interpretations of the accelerometry data by instrumenting and filming fur seals in captivity.

### Behavior categorization

The shaking behavior was easily identified by visually inspecting the accelerometry trace, while observing the captive animals made it possible to identify the grooming pattern that was associated with the shaking. The grooming behavior was a low frequency, irregular rolling pattern, typified by the animal staying on one side or the other for 1–10 seconds, turning over abruptly or gradually, remaining on the other side for another 1–10 seconds, and repeating this sequence while shaking every minute or so. Grooming maintains the fur for thermoregulation—and the time categorized as shaking to remove water and maintain the layer of air within the inner fur probably reflected the time (~10% of the time) needed to keep the fur in good thermoregulatory condition.

The most surprising result was the amount of time fur seals spent rolling at the surface (combined W-wave and sine wave), which accounted for nearly one-third of the entire foraging trip. It is unclear what specific purpose rolling at the surface may serve, though it may be used to scan for predators or prey. The rolling occurred in two forms that we named based on the graphic display of the accelerometer data—a sine-wave-roll (a 360° roll) and a W-roll (whereby the animal turns ~300° in one direction, then turns back 300° in the other direction). The rolling behavior, particularly the W-form, often continued among wild fur seals for hours at a time with little break, while captive animals did not sustain either of the rolling behaviors for more than a minute at a time. W-rolls were associated with faster swimming speeds and hence likely associated with transiting, while the sine-rolls appear to be associated with loitering.

The GPS-based speed analysis relative to the behaviors indicated that the prone position was associated with the fastest speeds—making it the most likely behavior associated with transiting. The St. Paul data were more informative as the fur seals from this island averaged a greater number of GPS fixes per day (19.1) than the Bogoslof seals (15.0 fixes per day), so calculated speeds were more likely to be associated with periods of distinct behaviors as the number of GPS fixes increased (Fig. 14). We expected diving to be associated with active swimming and high travel speeds, but did not expect the data to indicate shaking/grooming behaviors to be associated with high travel speeds. Shaking and grooming were associated with diving, but did not occur for extended periods at this time. Rather, shaking and grooming occurred most frequently during the first 24 hours of the fur seals leaving land (which may reflect the seals cleaning the dirt and fecal matter they may have gathered on their fur while on land). Thus, it appears that the speeds associated with shaking and grooming likely reflected speeds emanating from other associated behaviors.

### Filmed captive behavior

While it is difficult to unconditionally determine all behaviors from acceleration data, we found that many of the key behaviors that dominated the at-sea activity we recorded can be accurately categorized and successfully ground-truthed using captive animals. Some of the behaviors we classified for northern fur seals were previously known from direct observations, such as the jug handle resting position (where fur seals lie on one side and raise their hind flippers to touch the side flipper that extends out of the water). Shaking and various forms of rolling and grooming behaviors have also been commonly seen at the surface during the summer months in the waters near St. Paul and Bogoslof Islands. We were unable to associate flipper beats or porpoising with swimming at the surface. Flipper beats are easily recognizable during dives and were easily identifiable in the relatively calm pool at the aquarium. We presume that the flipper beats that occur while swimming at or near the surface in the wild are masked by signals from ocean waves, particularly surface chop.

### Behavior analyses

Because of the differences between the population trajectories of the two islands, as well as differences in trip lengths and the two different regions and strategies that animals use to forage from St. Paul Island, we expected to find differences in the relative amounts of behaviors between these ecological groupings. Specifically, we expected to find that the increasing population of Bogoslof fur seals that have short feeding trips would have an easier time foraging. However, it’s difficult to predict what an “easier time” would look like in relative amounts of behavior, but one might expect less time swimming and diving, and more time resting on the surface given that a satiated animal might be expected to dive less. The lack of differences may imply that behavioral allocations are more controlled by external factors. It may be, for example, that the amount of time that can be spent diving is limited by the amount of available darkness, (Fig. 13) and thus prey availability. It may also be that the behaviors of lactating northern fur seals as we measured them are not as flexible as we believed they should be, but are hardwired as part of a generalized successful foraging strategy that depends less on other behavioral choices, such as chosen foraging region (on-shelf vs. off-shelf). Given this data set and analysis, it appears that trip length is the only behavioral clue we have to foraging success.

## Supporting Information

S1 Fig Behavior ternary matrix for different islands. Ternary matrix for the 7 behaviors with the “mixed” behavior present in each ternary diagram. Proportions of each behavior are color and symbol coded by Island, Bogoslof or St. Paul. Note there is little differentiation in clumping of proportions by Island.
(TIF)

S2 Fig Behavior ternary matrix for different forager types. Ternary matrix for the 7 behaviors with the “mixed” behavior present in each ternary diagram. Proportions of each behavior are color and symbol coded by forager type, cathemeral or nocturnal. Note there is little differentiation in clumping of proportions by forager type.
(TIF)

S3 Fig Behavior ternary matrix for different regions. Ternary matrix for the 7 behaviors with the “mixed” behavior present in each ternary diagram. Proportions of each behavior are color and symbol coded by region, on-shelf or off-shelf. Note there is little differentiation in clumping of proportions by region.
(TIF)

S4 Fig Behavior boxplot matrix for different islands. Boxplot matrix of behavior log ratios for the 7 behaviors. Note that most of the boxplot notches overlap, indicating no significant behavioral differences between Bogoslof and St. Paul islands.
(TIF)

S5 Fig Behavior boxplot matrix for different forager types. Boxplot matrix of behavior log ratios for the 7 behaviors. Note that most of the boxplot notches overlap, indicating no significant behavioral differences between nocturnal and cathameral forager types.
(TIF)

S6 Fig Behavior boxplot matrix for different regions. Boxplot matrix of behavior log ratios for the 7 behaviors. Note that most of the boxplot notches overlap, indicating no significant behavioral differences between on-shelf and off-shelf regions.
(TIF)
